# Клинические рекомендации «Врожденная дисфункция коры надпочечников у детей» с комментариями

**DOI:** 10.14341/probl13767

**Published:** 2026-07-22

**Authors:** И. С. Чугунов, И. В. Копылова, А. А. Колодкина, Н. Ю. Калинченко, М. В. Воронцова, О. Б. Безлепкина, В. А. Петеркова, Н. Г. Мокрышева

**Affiliations:** Национальный медицинский исследовательский центр эндокринологии им. академика И.И. Дедова Россия; Endocrinology Research Centre Russian Federation; Национальный медицинский исследовательский центр эндокринологии им. академика И.И. Дедова; Медицинский научно-образовательный институт Московского государственного университета им. М.В. Ломоносова Россия; Endocrinology Research Centre; Medical Research and Educational Institute of Lomonosov Moscow State University Russian Federation

**Keywords:** врожденная дисфункция коры надпочечников, адреногенитальный синдром, дефицит 21-гидроксилазы, дети, CYP21A2, неонатальный скрининг, клинические рекомендации, congenital adrenal hyperplasia, 21-­hydroxylase deficiency, children, CYP21A2, neonatal newborn screening, Clinical Practice Guidelines

## Abstract

В клинических рекомендациях по врожденной дисфункции коры надпочечников у детей кратко и структурировано изложены основные сведения по эпидемиологии, этиологии, патогенезу, методам диагностики всех форм заболевания. Представлены схемы заместительной терапии глюкокортикоидными и минералокортикоидными препаратами и протоколы наблюдения за пациентами разных возрастных категорий. Настоящие клинические рекомендации разработаны и одобрены экспертным сообществом детских эндокринологов Российской ассоциации эндокринологов и утверждены советом Министерства здравоохранения РФ. В основу клинических рекомендаций включены систематические обзоры, мета-анализы, оригинальные статьи и научные работы по данной патологии в Российской Федерации и других странах. В статье обновлены комментарии и разъяснения авторов по наиболее важным аспектам диагностики и лечения врожденной дисфункции коры надпочечников в детском возрасте.

## Abstract (en)

The clinical practice guidelines for congenital adrenal hyperplasia (CAH) in children clearly and systematically present the key information on the epidemiology, etiology, pathogenesis, and diagnostic methods for all forms of the disease. Protocols for glucocorticoid and mineralocorticoid replacement therapy, as well as follow-up regimens for patients in different age groups, are provided. These clinical guidelines were developed and approved by the expert community of pediatric endocrinologists from the Russian Association of Endocrinologists and endorsed by the council of the Russian Federation Ministry of Health. The guidelines are based on systematic reviews, meta-analyses, original articles, and scientific studies on this pathology conducted in the Russian Federation and other countries. This article provides updated author comments and clarifications on the most critical aspects of the diagnosis and treatment of congenital adrenal hyperplasia in childhood.

## СПИСОК СОКРАЩЕНИЙ

## ТЕРМИНЫ И ОПРЕДЕЛЕНИЯ

Врожденная дисфункция коры надпочечников (адреногенитальный синдром, врожденная гиперплазия надпочечников) — группа заболеваний с аутосомно-рецессивным типом наследования, в основе которых лежит дефект одного из ферментов или транспортных белков, принимающих участие в биосинтезе кортизола в коре надпочечников

Преждевременное адренархе — изолированное появление лобкового оволосения у девочек до 8 лет и у мальчиков до 9 лет.

Гонадотропин-зависимое преждевременное половое развитие — появление вторичных половых признаков у девочек до 8 лет и у мальчиков до 9 лет, обусловленное преждевременной активацией гипоталамо-гипофизарной системы.

Определение спектра стероидов методом ТМС — количественное определение основных стероидных гормонов и их метаболитов в сыворотке крови методом тандемной хромато-масс-спектрометрией (LC-MS/MS).

Уровень достоверности доказательств — степень уверенности в том, что найденный эффект от применения медицинского вмешательства является истинным

Уровень убедительности рекомендаций — степень уверенности в достоверности эффекта вмешательства и в том, что следование рекомендациям принесет больше пользы, чем вреда в конкретной ситуации

Сложившаяся клиническая практика — (англ. good practice point, GPP) используется для тезисов-рекомендаций, относящихся к сбору жалоб и анамнеза пациента, физикальному осмотру пациента, а также характеризующих организацию медицинской помощи (организацию медицинского процесса) в случае, если отсутствуют доказательства, полученные на основании результатов систематического поиска и отбора клинических исследований.

## 1. КРАТКАЯ ИНФОРМАЦИЯ ПО ЗАБОЛЕВАНИЮ ИЛИ СОСТОЯНИЮ (ГРУППЕ ЗАБОЛЕВАНИЙ ИЛИ СОСТОЯНИЙ)

## 1.1 Определение заболевания или состояния (группы заболеваний или состояний)

Врожденная дисфункция коры надпочечников (адреногенитальный синдром, врожденная надпочечниковая гиперплазия) — группа заболеваний с аутосомно-рецессивным типом наследования, в основе которых лежит дефект одного из ферментов или транспортных белков, принимающих участие в биосинтезе кортизола в коре надпочечников.

## 1.2 Этиология и патогенез заболевания или состояния (группы заболеваний или состояний)

В основе всех форм ВДКН лежат патологические вариантные замены в генах, отвечающих за синтез ферментов или коферментов стероидогенеза, а также гена STAR, кодирующего белок, который участвует в транспорте холестерина внутрь митохондрий [1–8]. Для всех форм ВДКН доказан аутосомно-рецессивный тип наследования, т.е. для проявления заболевания необходимо наличие мутаций на обоих аллелях. Также при всех формах заболевания имеется генотип-фенотипическая корреляция, при которой выраженность клинических проявлений напрямую зависит от степени нарушения функции фермента. Таким образом, описаны классические формы заболевания с дебютом клинической картины в ранний неонатальный период, и неклассические стертые формы заболевания, когда клиническая картина развивается в более позднем детском возрасте, в период пубертата или даже у молодых взрослых [5][9][10]. При наличии компаунд-гетерозиготных мутаций, клиническую картину определяет менее «тяжелая» мутация.

При всех формах ВДКН отмечается дефицит кортизола, что по механизму отрицательной обратной связи приводит к повышению уровня АКТГ и гиперплазии надпочечников. В результате стимуляции надпочечников происходит избыточное накопление стероидов, предшествующих ферментативному блоку. Клиническая картина каждой формы ВДКН обусловлена дефицитом гормонов, синтез которых невозможен при данном ферментативном блоке, и избытком предшественников.

## 1.3 Эпидемиология заболевания или состояния (группы заболеваний или состояний)

Самой частой формой ВДКН, на которую приходится более 90% больных, является дефицит 21‑гидроксилазы. Распространенность дефицита 21‑гидроксилазы в мире, рассчитанная по данным неонатального скрининга, составляет 1 случай на 14 000 живых новорожденных [11]. В России по данным неонатального скрининга частота классических форм дефицита 21‑гидроксилазы составляет 1 случай на 9638 живых новорожденных [12]. Распространенность неклассической формы дефицита 21‑гидроксилазы в мире составляет 0,1–0,2%, среди евреев-ашкенази данная форма встречается значительно чаще — 1–2%. Минимальная распространенность неклассической формы дефицита 21‑гидроксилазы, рассчитанная на основании определения гетерозиготного носительства двух наиболее частых мутаций в гене CYP21A2 по формуле Харди-Вайнберга на примере Московской области, составила 1 случай на 2206 новорожденных [13].

Второй по распространенности формой ВДКН является дефицит 11в‑гидроксилазы. В мире данная форма ВДКН встречается в 10 раз реже, чем классические формы дефицита 21‑гидроксилазы. Распространенность дефицита 11в‑гидроксилазы в России не изучена. Остальные формы ВДКН встречаются значительно реже. Для некоторых редких форм ВДКН характерна высокая частота встречаемости в отдельных популяциях, что объясняется как частотой близкородственных браков, так и эффектом основателя. Так описана высокая распространенность дефицита Зв‑гидроксистероиддегидрогеназы, которая составляет 1 случай на 22 679 живых новорожденных в Республике Осетия [14].

Учитывая подавляющее преобладание дефицита 21‑гидроксилазы среди всех форм ВДКН, основная часть клинических рекомендаций относится к диагностике и лечению именно этой формы.

## 1.4 Особенности кодирования заболевания или состояния (группы заболеваний или состояний) по Международной статистической классификации болезней и проблем, связанных со здоровьем

## 1.5 Классификация заболевания или состояния (группы заболеваний или состояний)

На сегодняшний день известно 7 форм врожденной дисфункции коры надпочечников (ВДКН):

Внутри каждой формы выделяют классические и неклассические варианты, в зависимости от тяжести и сроков клинических проявлений. Деление на классическую и неклассическую форму обусловлено разной степенью снижения активности фермента, что определяется конкретной мутацией.

При дефиците 21‑гидроксилазы выделяют 3 клинические формы: две классические — сольтеряющую, вирильную и неклассическую форму заболевания. При нулевой активности фермента определяется тотальный дефицит глюко- и минералокортикоидов, что приводит к сольтеряющему состоянию в период новорожденности и различной степени выраженности вирилизации наружных гениталий у девочек. При остаточной активности фермента от 1 до 5% развивается вирильная форма заболевания без нарушения минералокортикоидной функции, но приводящая к внутриутробной вирилизации плода. При активности фермента более 5% вирилизация проявляется только после рождения в виде преждевременного адренархе в 4–5 лет, ускорения роста и костного возраста [12][15][16].

## 1.6 Клиническая картина заболевания или состояния (группы заболеваний или состояний)

Характерный для всех форм ВДКН дефицит кортизола проявляется склонностью к гипогликемическому синдрому, особенно выраженной у грудных детей и в стрессовых ситуациях. Специфическим симптомом дефицита кортизола является гиперпигментация кожи вследствие высокого уровня проопиомеланокортина и его производных.

В зависимости от уровня ферментативного блока все формы ВДКН можно разделить на 2 группы: при дефиците фермента на начальных этапах стероидогенеза (дефицит 11а‑гидроксилазы, дефект STAR-протеина, дефицит 17а‑гидроксилазы) имеется блок на пути синтеза андрогенов как в надпочечниках, так и в гонадах, что приводит к недостаточной маскулинизации наружных половых органов у плодов с кариотипом 46XY; при дефиците ферментов на более поздних этапах стероидогенеза, протекающих только в надпочечниках (дефицит 21‑гидроксилазы, дефицит 11в‑гидроксилазы), происходит накопление надпочечниковых андрогенов, что приводит к внутриутробной вирилизации плодов с кариотипом 46ХХ. При дефиците 3в‑гидроксистероиддегидрогеназы при рождении отмечается неправильное строение наружных половых органов как при кариотипе 46ХХ, так и при кариотипе 46XY, поскольку имеется блок на пути синтеза тестостерона, что приводит к недостаточной маскулинизации у мальчиков, но накапливается в избытке слабые андрогены (ДГЭА), что вызывает небольшую вирилизацию у девочек. Также, неправильное строение наружных гениталий как у мальчиков, так и у девочек может наблюдаться при дефиците оксидоредуктазы.

В постнатальном периоде формы, сопровождающиеся избытком надпочечниковых андрогенов, проявляются ускорением роста, ускорением костного возраста и преждевременным адренархе. Наоборот, при формах ВДКН с нарушением синтеза половых стероидов, в подростковом возрасте развивается гипергонадотропный гипогонадизм как у мальчиков, так и у девочек.

В отношении нарушения минералокортикоидной функции надпочечников все формы ВДКН также можно разделить на 2 группы: протекающие с дефицитом минералокортикоидов (дефицит 11а‑гидроксилазы, дефект STAR-протеина, дефицит 3в‑гидроксистероиддегидрогеназы, дефицит 21‑гидроксилазы, дефицит оксидоредуктазы), протекающие с избытком минералокортикоидов (дефицит 11в‑гидроксилазы, дефицит 17а‑гидроксилазы). Клиническими проявлениями дефицита минералокортикоидов являются признаки синдрома потери соли: тошнота, неукротимая рвота, снижение АД, потеря веса, жидкий стул и обезвоженность организма. Для дефицита минералокортикоидов характерны электролитные изменения в виде гиперкалиемии и гипонатриемии, гормональным маркером является повышение уровня ренина в крови. Избыток минералокортикоидов ведет к формированию стойкой артериальной гипертензии, сопровождающейся выраженной гипокалиемией с характерными симптомами мышечной слабости, полиурии и полидипсии, гормональным маркером является сниженный уровень ренина плазмы. Клиническая картина всех форм ВДКН представлена в таблице 1.

**Table:** Таблица 1. Клинико-гормональные характеристики различных форм ВДКН.

Форма ВДКН	ген	МК	ПС	Клиническая картина	Гормональный маркер
Синдром потери соли/АГ	46XYСтроение гениталий/ половое развитие	46XXСтроение гениталий/ половое развитие
Дефект StAR протеина (липоидная гиперплазия надпочечников)	STAR8p11.2	↓	↓	Синдром потери соли	Женское/ гипогонадизм	женское / гипогонадизм	нет
Дефицит 11а‑гидроксилазы (дефицит 20,22-десмолазы)	CYP11A115q23-q24	↓	↓	Синдром потери соли	Женское/ гипогонадизм	женское / гипогонадизм	нет
Дефект 17 а‑гидроксилазы/17,20-лиазы	CYP1710q24.3	↑	↓	АГ	Промежуточное, ближе к женскому/ гипогонадизм	женское/ гипогонадизм	Дезоксикортикостерон, кортикостерон
Дефект 3в‑гидроксистероиддегидрогеназы	HSD3B21p13.1	↓	↓	Синдром потери соли	Промежуточное с андрогенизацией в пубертате	Промежуточное ближе кженскому	А5-стероиды: прегненолон, 17ОН-прегненолон, ДГЭА
Дефицит 21‑гидроксилазы	CYP21A26p21.3	↓ или норма	↑	Синдром потери соли/N	мужское	промежуточное	17ОНР
Дефицит 11в‑гидроксилазы	CYP11B18q21	↑	↑	АГ	мужское	промежуточное	11-ДОК,11‑дезоксикортикостерон
Дефицит оксидоредуктазы	POR7q11.2	↓ или норма	↓	нет	промежуточное	промежуточное	17ОНР, прогестерон, 17ОН-прегненолон

## 2. ДИАГНОСТИКА ЗАБОЛЕВАНИЯ ИЛИ СОСТОЯНИЯ (ГРУППЫ ЗАБОЛЕВАНИЙ ИЛИ СОСТОЯНИЙ), МЕДИЦИНСКИЕ ПОКАЗАНИЯ И ПРОТИВОПОКАЗАНИЯ К ПРИМЕНЕНИЮ МЕТОДОВ ДИАГНОСТИКИ

Для различных форм ВДКН характерно разнообразие клинических проявлений. Симптомы заболевания определяются дефицитом одних и избытком других классов стероидов, обусловленных конкретным ферментативным блоком. Основной лабораторный метод диагностики всех форм ВДКН — это определение различных кортикостероидов в сыворотке крови. При классических вариантах заболевания стероидные соединения, предшествующие ферментативному блоку, повышены в десятки раз, что не вызывает затруднений в постановке диагноза. Следует помнить, что все стероидные соединения имеют схожую химическую структуру, что обуславливает значительный перекрест между ними при определении их концентрации иммуноферментным или радиоиммунологическим методом. В настоящее время максимальной информативностью обладает метод мультистероидного анализа с предшествующим разделением всех стероидов с помощью хроматографии или масс- спектрометрии. При использовании данного метода можно не только определить точную концентрацию стероидных соединений, но и оценить соотношение предшественников и продуктов различных ферментов. В таблице 1 представлены клинические особенности и гормональные маркеры каждой из форм ВДКН.

## 2.1 Жалобы и анамнез

При классических формах заболевания дебют клинической картины, как правило, происходит в неонатальный период в виде неправильного строения наружных гениталий и сольтеряющего синдрома. Исключение составляют гипертонические формы ВДКН, при которых повышение артериального давления, как правило, начинается в раннем детстве.

При неклассических (стертых) формах ВДКН клинические проявления могут дебютировать как в периоде детства, так и в пубертатном возрасте, а иногда диагноз устанавливается уже во взрослом состоянии.

Уровень убедительности рекомендаций C (уровень достоверности доказательств — 5).

Уровень убедительности рекомендаций C (уровень достоверности доказательств — 5)

Уровень убедительности рекомендаций С (уровень достоверности доказательств — 4)

Уровень убедительности рекомендаций B (уровень достоверности доказательств — 2)

## 2.2 Физикальное обследование

Физикальное обследование новорожденных в первую очередь подразумевает оценку строения наружных гениталий по шкале Прадера и выявление признаков сольтеряющего синдрома: обезвоживание, снижение веса, срыгивания, упорная рвота.

Осмотр детей должен включать в себя оценку общего состояния, полового статуса и анализ антропометрических данных (измерение роста и массы тела с оценкой стандартных отклонений от популяционных норм).

Уровень убедительности рекомендаций C (уровень достоверности доказательств — 5)

Комментарии: шкала Прадера представлена в приложении (Рисунок 3).

Уровень убедительности рекомендаций C (уровень достоверности доказательств — 5)

Комментарии: клинические проявления синдрома потери соли в виде срыгиваний, потери веса, жидкого стула, снижения артериального давления и признаков эксикоза возникают чаще на 10–20 день жизни, но могут наблюдаться и значительно раньше. Электролитные изменения в виде гиперкалиемии и гипонатриемии выявляются еще на доклинической стадии и могут фиксироваться в первые дни жизни.

Комментарии авторов: умеренные электролитные нарушения могут отмечаться у новорожденных с классическими формами врожденной дисфункцией коры надпочечников с первых дней жизни. Выраженная гипонатриемия (натрий <130 ммоль/л) и гиперкалиемия (калий >6 ммоль/л) обычно наблюдаются на второй неделе жизни [25].

Уровень убедительности рекомендаций C (уровень достоверности доказательств — 4)

Комментарии: одной из отличительных черт врожденной дисфункции коры надпочечников от идиопатического преждевременного адренархе является ускорение роста более 2 SD и опережение костного возраста более чем на 2 года от паспортного.

Уровень убедительности рекомендаций C (уровень достоверности доказательств — 4)

Комментарии: диссоциация между стадией полового развития и допубертатным размером яичек может свидетельствовать в пользу избытка андрогенов надпочечникового генеза.

## 2.3 Лабораторные диагностические исследования

## 2.3.1. Диагностика дефицита 21‑гидроксилазы в рамках неонатального скрининга

Уровень убедительности рекомендаций C (уровень достоверности доказательств — 4)

Комментарии: в России неонатальный скрининг проводится с середины 2006 г., что привело более чем к двукратному увеличению выявляемости данного заболевания. Первоначально процедура скрининга предусматривала забор крови у доношенных новорожденных на 4‑е сутки жизни, у недоношенных — на 7–10‑е сутки, с последующим определением уровня 17‑ОНР с использованием специализированных тест-систем. В 2023 г. были внесены изменения в программу: для неонатального и расширенного неонатального скрининга на врожденные и (или) наследственные заболевания образцы крови из пятки новорожденного берут через 3 часа после кормления в возрасте 24–48 часов у доношенных и на 7‑е сутки (144–168 часов) жизни у недоношенных детей.

Пороговые значения зависят от массы тела при рождении и устанавливаются отдельно для каждой лаборатории [11]. При положительном результате первичного скрининга информация направляется в поликлинику по месту жительства ребенка, после чего проводится повторный забор крови для ретестирования.

По данным литературы, у недоношенных новорожденных более информативной считается градация диагностических уровней 17‑ОНР с учетом гестационного возраста, а не массы тела при рождении [30].

Уровень убедительности рекомендаций В (уровень достоверности доказательств — 3)

Комментарии: положительная предсказательная ценность первого этапа неонатального скрининга составляет от 1 до 5%, т. е. среди всех положительных результатов только от 1 до 5 детей из 100 положительных образцов действительно имеют ВДКН, в остальных случаях это ложноположительные результаты. Учитывая большие экономические и эмоциональные затраты, в связи с большим количеством ложноположительных случаев было предложено проводить повторное тестирование с использованием тандемной масс-спектрометрии (ТМС). По данным немецких авторов, использование ТМС и определение расчетного показателя (сумма уровней 17ОН-прогестерона и 21‑дезоксикортизола, деленная на уровень кортизола) повышает положительную предсказательную ценность до 100% [36]. У детей без дефицита 21‑гидроксилазы данное соотношение <0,07, напротив, у детей с дефицитом 21‑гидроксилазы >0,53. Помимо выявления дефицита 21‑гидроксилазы, ТМС позволяет диагностировать и редкие формы ВДКН [37].

Уровень убедительности рекомендаций C (уровень достоверности доказательств — 5)

Комментарии: учитывая возможность ложноположительных результатов неонатального скрининга, а также тот факт, что неонатальный скрининг позволяет выявить небольшой процент детей с неклассической формой дефицита 21‑гидроксилазы, которым не требуется назначение лечения, всем детям с положительным результатом неонатального скрининга необходим осмотр врача-детского эндокринолога. Неправильное строение наружных половых органов в сочетании с положительным результатом скрининга однозначно позволяет установить диагноз у девочек и назначить терапию. В то же время всем мальчикам и девочкам с правильным строением наружных гениталий может потребоваться дополнительное обследование.

Уровень убедительности рекомендаций С (уровень достоверности доказательств — 5)

Комментарии: выявление гипонатриемии в сочетании с гиперкалиемией позволяет заподозрить сольтеряющую форму дефицита 21‑гидроксилазы. Необходимо отметить, что одной из целей неонатального скрининга является диагностика заболевания до развития клинической картины сольтеряющего синдрома, которая как правило дебютирует на 2–3 неделях жизни. К моменту получения результатов скрининга (10–14 сутки жизни ребенка) даже при отсутствии клинической картины синдрома потери соли у пациента уже имеются электролитные нарушения.

Комментарии авторов: несмотря на то, что дефект биосинтеза альдостерона клинически проявляется только при сольтеряющей форме, субклинический дефицит альдостерона присутствует при всех формах дефицита 21‑гидроксилазы. Умеренные электролитные нарушения могут наблюдаться в раннем неонатальном периоде и у детей с вирильной формой ВДКН [42].

Уровень убедительности рекомендаций C (уровень достоверности доказательств — 4)

Комментарии: при пограничных значениях 17ОНР («серая зона») в рамках неонатального скрининга и при последующем гормональном обследовании по данным зарубежной литературы рекомендуется проводить стимулирующий тест с препаратами из АТХ-группы АКТГ (H01AA), что является «золотым стандартом» диагностики ВДКН. При сомнительных результатах пробы или невозможности ее проведения рекомендовано молекулярно-генетическое исследование гена CYP21A2.

Дефект 21‑гидроксилазы обусловлен многочисленными мутациями гена CYP21A2, кодирующего данный этот фермент. Ген расположен на коротком плече 6‑й хромосомы рядом с главным комплексом гистосовместимости (HLA). В непосредственной близости с геном CYP21A2 расположен псевдоген CYP21P. Эти гены высокогомологичны, однако, участвует в транскрипции 21‑гидроксилазы только CYP21A2, имеющий в своей структуре 10 транскрибируемых экзонов и 9 интронов, а CYP21P является псевдогеном, т.к. содержит ряд мутаций, из-за которых кодируемый ими белок полностью лишен активности. Тандемная организация высокогомологичных генов создает предпосылки к частым рекомбинациям между ними в виде замещения большого фрагмента гена CYP21A2 аналогичным фрагментом псевдогена или переноса маленьких фрагментов псевдогена в активный ген (генная конверсия). Подобные мутации могут приводить к полной или частичной потере ферментативной активности 21‑гидроксилазы. В настоящее время описаны более трехсот мутаций CYP21A2, приводящих к дефекту 21‑гидроксилазы, но 85% всех случаев приходится на 12 частых мутаций, перешедших из псевдогена. Имеется генотип-фенотипическая корреляция между видом мутации и клиническим вариантом течения заболевания [3].

На первом этапе молекулярно-генетической диагностики рекомендуется проводить поиск частых мутаций в гене CYP21A2. Выявление гомозиготной мутации или двух гетерозиготных мутаций позволяет поставить диагноз. При обнаружении одной гетерозиготной мутации или при отсутствии частых мутаций проводится полное секвенирование гена CYP21A2 [3].

## 2.3.2. Диагностика дефицита 21‑гидроксилазы вне процедуры неонатального скрининга

Уровень убедительности рекомендаций C (уровень достоверности доказательств — 5)

Комментарии: основным критерием гормональной диагностики недостаточности 21‑гидроксилазы является повышение уровня 17‑ОНР в сыворотке крови. Содержание 17‑ОНР при классических формах заболевания более чем в 10 раз превышает нормативные для возраста ребенка показатели.

Комментарии авторов: при неклассической форме ВДКН обычно отмечается умеренное повышение 17-OHP. При базальных показателях 17ОНР более 30 нмоль/л (10 нг/мл), диагноз ВДКН считается подтвержденным [20].

Уровень убедительности рекомендаций C (уровень достоверности доказательств — 5)

Комментарии: следует помнить, что все стероидные соединения имеют схожую химическую структуру, что обуславливает значительный перекрест между ними при определении их концентрации. В настоящее время максимальной информативностью обладает метод мультистероидного анализа с предшествующим разделением всех стероидов с помощью хроматографии или масс-спектрометрии. При использовании данного метода можно не только определить точную концентрацию стероидных соединений, но и оценить соотношение предшественников и продуктов различных ферментов. Выявление в пробе крови повышенного уровня 21‑дезоксикортизола является специфическим маркером дефицита 21‑гидроксилазы.

Уровень убедительности рекомендаций C (уровень достоверности доказательств — 4)

Комментарии: на первом этапе осуществляется поиск частых мутаций в гене CYP21A2. Выявление гомозиготной мутации или двух гетерозиготных мутаций позволяет поставить диагноз. При обнаружении одной гетерозиготной мутации или при отсутствии частых мутаций проводится полное секвенирование гена CYP21A2.

Уровень убедительности рекомендаций C (уровень достоверности доказательств — 5)

Комментарии: при врожденной дисфункции коры надпочечников высокие дозы дексаметазона** приводят к значимому снижению уровня андрогенов, тогда как при андрогенпродуцирующей опухоли уровень андрогенов не меняется на фоне введения дексаметазона**. Протокол проведения пробы идентичен малой дексаметазоновой пробе, используемой при диагностике гиперкортицизма: до пробы берется анализ крови для определения андрогенов (исследование уровня 17‑гидроксипрогестерона, дегидроэпиандростерона сульфата, общего тестостерона в крови) и кортизола, затем в течение 2 суток принимается дексаметазон** в дозе по 0,5 мг х 4 раза в сутки (4 мг на пробу) и на третий день утром берется анализ крови на содержание андрогенов (исследование уровня 17‑гидроксипрогестерона, дегидроэпиандростерона сульфата, общего тестостерона в крови) и исследование уровня кортизола крови.

Уровень убедительности рекомендаций C (уровень достоверности доказательств — 4)

2.3.3 Диагностика редких форм врожденной дисфункции коры надпочечников

Уровень убедительности рекомендаций С (уровень достоверности доказательств — 5)

Комментарии: для липоидной гиперплазии коры надпочечников, для дефицита 11а- гидроксилазы, для дефицита 17а‑гидроксилазы, дефицита 3в‑гидроксистероди- дегидрогеназы и для дефицита оксидоредуктазы характерно неправильное или женское строение наружных гениталий при кариотипе 46 XY, и наоборот для дефицита 11в‑гидроксилазы характерно неправильное строение наружных половых органов при кариотипе 46ХХ, для дефицита 3в‑гидроксистероиддегидрогеназы и для дефицита оксидоредуктазы при кариотипе 46ХХ возможна небольшая вирилизация наружных половых органов.

Уровень убедительности рекомендаций C (уровень достоверности доказательств — 5)

Комментарии: проведение мультистероидного анализа у детей с нарушением формирования пола позволяет диагностировать все классические варианты нарушений стероидогенеза. Для каждой формы ВДКН характерно повышение определенных стероидов, которые указаны в таблице 1.

Уровень убедительности рекомендаций C (уровень достоверности доказательств — 5)

Комментарии: развитие сольтеряющего синдрома у новорожденных может привести к тяжелым последствиям вплоть до летального исхода, поэтому всем новорожденным с неправильным строением половых органов, причиной чего могут быть редкие формы врожденной дисфункции коры надпочечников, необходимо исследовать уровень калия и натрия в крови. Гиперкалиемия в сочетании с гипонатриемией являются признаками дефицита минералокортикоидов и требуют немедленного назначения терапии.

Уровень убедительности рекомендаций C (уровень достоверности доказательств — 5)

Комментарии: повышение уровня АКТГ у новорожденного с неправильным строением наружных гениталий позволяет диагностировать один из вариантов врожденной дисфункции коры надпочечников. Повышение уровня ренина крови в сочетании с низким уровнем альдостерона отмечаются при формах ВДКН, протекающих с дефицитом минералокортикоидов (липоидная гиперплазия надпочечников, дефицит 11а‑гидроксилазы (дефицит 20,22-десмолазы), дефицит 3 в‑гидроксистероиддегидрогеназы).

Уровень убедительности рекомендаций C (уровень достоверности доказательств — 5)

Комментарии: наличие низкорениновой артериальной гипертензии в сочетании с симптомами гиперандрогении являются характерными для дефицита 11в‑гидроксилазы, что подтверждается высоким уровнем 11‑дезоксикортизола и 11‑дезоксикортикостерона в сочетании с высокими надпочечниковыми андрогенами.

Уровень убедительности рекомендаций C (уровень достоверности доказательств — 5)

## 2.4 Инструментальные диагностические исследования

Уровень убедительности рекомендаций C (уровень достоверности доказательств — 5)

Уровень убедительности рекомендаций C (уровень достоверности доказательств — 5)

Уровень убедительности рекомендаций C (уровень достоверности доказательств — 5)

Комментарии: у девочек с нарушениями менструального цикла на фоне аменореи может определяться умеренно повышенный уровень 17‑гидроксипрогестерона. Выявление на УЗИ увеличенных яичников (более 12 мл) может являться одним из свидетельств в пользу овариального генеза гиперандрогении.

Комментарии авторов: синдром поликистозных яичников имеет схожую клиническую картину с неклассической формой врожденной дисфункцией коры надпочечников. Наоборот, у пациенток с врожденной дисфункцией коры надпочечников, преимущественно с классическими формами и при неудовлетворительной компенсации заболевания, может формироваться синдром поликистозных яичников. В патогенезе формирования поликистозных яичников при ВДКН участвуют инсулинорезистентность, ожирение и избыточная секреция андрогенов [57].

Уровень убедительности рекомендаций C (уровень достоверности доказательств — 5)

Комментарии: отсутствие матки у новорожденного с кариотипом 46XY при неправильном или женском типе строения наружных гениталий может наблюдаться при врожденной дисфункции коры надпочечников (при липоидной гиперплазии надпочечников, при дефиците 11а‑гидроксилазы, дефиците 17а‑гидроксилазы, дефиците 3в‑гидроксистероиддегидрогеназы).

## 2.5 Иные диагностические исследования

Уровень убедительности рекомендаций C (уровень достоверности доказательств — 5)

## 3. ЛЕЧЕНИЕ, ВКЛЮЧАЯ МЕДИКАМЕНТОЗНУЮ И НЕМЕДИКАМЕНТОЗНУЮ ТЕРАПИИ, ДИЕТОТЕРАПИЮ, ОБЕЗБОЛИВАНИЕ, МЕДИЦИНСКИЕ ПОКАЗАНИЯ И ПРОТИВОПОКАЗАНИЯ К ПРИМЕНЕНИЮ МЕТОДОВ ЛЕЧЕНИЯ

## 3.1 Консервативное лечение

Основой терапии при всех формах ВДКН является назначение глюкокортикоидов, которые позволяют заместить дефицит кортизола и тем самым подавить избыточную секрецию АКТГ. В результате снижается продукция надпочечниками тех стероидов, которые в избытке синтезируются при данном ферментативном блоке.

Уровень убедительности рекомендаций С (уровень достоверности доказательств — 4)

Уровень убедительности рекомендаций C (уровень достоверности доказательств — 4)

Комментарии: существуют различные медикаментозные препараты, обладающие глюкокортикоидной активностью: гидрокортизон**, преднизолон**, кортизон, дексаметазон**. Синтетические глюкокортикоиды пролонгированного действия (преднизолон**, дексаметазон**) оказывают негативное влияние на процессы роста. Для детей с открытыми зонами роста, особенно младшего возраста, наиболее оптимальными препаратами следует считать таблетированные формы гидрокортизона**. Первоначальная суточная доза гидрокортизона**, необходимая для подавления избыточной секреции АКТГ у детей первого года жизни, может достигать 20 мг/м². У детей старше года суточная доза гидрокортизона** в среднем должна составлять 10–15 мг/м². Препарат дается три раза в сутки в равных дозах каждые 8 часов.

Комментарии авторов: В течение первых месяцев жизни, когда уровень надпочечниковых андрогенов достигает целевого диапазона, следует снижать дозировку гидрокортизона** до стандартной заместительной (10–15 мг/м²/сут) [65]. Необходимо помнить, что попытки полностью нормализовать уровень 17OHP чаще приводят к клинической картине передозировки глюкокортикоидами.

В последнее время в зарубежной литературе появились сведения о возможном преимуществе 4-х кратного приема гидрокортизона** у детей с ВДКН [66]. Метаболизм кортизола вариабелен у различных людей, в связи с особенностями распределения и клиренса этого гормона. Авторы исследований отмечают, что 4-кратный прием гидрокортизона** лучше имитирует циркадный ритм кортизола и позволяет избежать его сверхфизиологических концентраций с последующими длительными периодами гипокортизолемии [67]. Работы по сравнению режимов дозирования выполнены на небольших когортах пациентов, и в настоящее время рекомендации по 4-х кратному приему гидрокортизона** не имеют достаточной доказательной базы. Тем не менее, в отдельных случаях возможно увеличение частоты приема препарата [68].

Уровень убедительности рекомендаций C (уровень достоверности доказательств — 4)

Комментарии: у детей, достигших конечного роста и/или с зонами роста, близкими к закрытию, возможно применение глюкокортикоидов пролонгированного действия, оказывающих более выраженный АКТГ-подавляющий эффект. Суточная доза этих препаратов должна соответствовать эквивалентной дозе гидрокортизона** 10–15 мг/м² в день (преднизолон** 5–7,5 мг/сут в 2 приема, дексаметазон** 0,25–0,5 мг/сут в 1–2 приема). Для максимального подавления секреции АКТГ 1/3 суточной дозы назначается в утренние часы (в 07:00) и 2/3 дозы — перед сном (в 23:00).

Уровень убедительности рекомендаций B (уровень достоверности доказательств — 4)

Комментарии: при сольтеряющей форме ВДКН необходима терапия минералокортикоидом флудрокортизоном**, который назначается в дозе 0,05–0,15 мг/сут в 1–2 приема (в 06:00–07:00 и в 16:00–17:00). У детей грудного возраста потребность в минералокортикоидах выше и может достигать 0,3 мг/сут (суточную дозу рекомендуется разделить на 2–3 приема). У детей без клинических проявлений сольтеряющего синдрома может отмечаться субклинический дефицит минералокортикоидов, критерием которого является повышенный уровень ренина. В таких случаях тоже показано назначение флудрокортизона**. Детям грудного возраста показано дополнительное введение в пищевой рацион поваренной соли в количестве 0,1–0,2 г на кг веса.

Комментарии авторов: для расчета количества поваренной соли и перевода из миллимолей в граммы следует использовать формулу:

G=ммоль*M(г/моль)/1000,

где:

- G — масса в граммах

- ммоль — заданное количество в миллимолях

- М — молярная масса вещества (M поваренной соли (хлорид натрия) = 58,5 г/моль).

Дополнительная дотация поваренной соли может не требоваться при использовании больших доз флудрокортизона**. Значимое повышение ренина плазмы (более 10нг/мл/час для активности ренина плазмы или более 100мкМЕ/мл для прямого ренина) у детей старше 3 месяцев без клинических проявлений синдрома потери соли может указывать на субклиинический дефицит минералокортикоидов.

## 3.2 Хирургическое лечение

Уровень убедительности рекомендаций C (уровень достоверности доказательств — 5)

Комментарии авторов: в настоящее время при хирургической коррекции наружных половых органов у девочек с различной степенью вирилизации описаны два основных подхода: одноэтапная и двухэтапная феминизирующая пластика. Одноэтапное вмешательство предполагает одновременное выполнение операций на клиторе с сохранением сосудисто-нервного пучка и интроитопластики. Двухэтапный подход предусматривает выполнение клиторопластики и, при необходимости, рассечения урогенитального синуса в раннем возрасте с последующей интроитопластикой в пубертатном возрасте. Вопросы оптимальных сроков и объёма хирургической коррекции, а также отдалённых косметических и функциональных результатов остаются предметом дальнейших исследований.

Уровень убедительности рекомендаций C (уровень достоверности доказательств — 5)

## 3.3 Иное лечение

Одним из осложнений длительной декомпенсации тех форм ВДКН, которые протекают с избыточным синтезом надпочечниковых андрогенов (дефицит 21‑гидроксилазы и дефицит 11в‑гидроксилазы) является прогрессия костного возраста. При достижении пубертатных показателей костного возраста у детей может развиться гонадотропинзависимое преждевременное половое развитие. Для подтверждения преждевременной активации гипоталамо-гипофизарно-гонадной системы проводится проба с аналогами гонадотропинрилизинг гормона. В случае неудовлетворительного ростового прогноза (предполагаемый конечный рост менее 150 см у женщин и менее 160 см у мужчин) возможно назначение аналогов гонадотропинрилизинг гормона (код ATX L02AE) по стандартной схеме [74]. На фоне блокирования полового развития у детей, как правило, снижается скорость роста. В таких случаях возможно дополнительное применение препаратов гормона роста с целью улучшения ростового прогноза [12][75].

Уровень убедительности рекомендаций В (уровень достоверности доказательств — 3)

Комментарии: аналоги гонадотропин-рилизинг гормона (код ATX L02AE) (#трипторелин** в дозе 3,75 мг в/м 1 раз в 28 дней или 11,25 мг в/м 1 раз в 12 недель, #лейпрорелин** в дозе 7,5 мг в/м 1 раз в 28 дней или 11,25 мг в/м 1 раз в 12 недель — лечение осуществляется в соответствии с соответствующими клиническими рекомендациями «Преждевременное половое развитие» [78]. Препараты гормона роста (соматропин** в дозе 0,035 мг на кг/сут п/к в вечерние часы ежедневно) также могут быть назначены в случае неудовлетворительного ростового прогноза (предполагаемый конечный рост менее 150 см у женщин и менее 160 см у мужчин).

Уровень убедительности рекомендаций C (уровень достоверности доказательств — 5)

Комментарии: при сохраняющейся артериальной гипертензии на фоне терапии глюкокортикоидами при гипертонических формах ВДКН показано назначение дополнительной гипотензивной терапии в случае невозможности эскалации дозы глюкокортикоидов [79][80]. Предпочтение отдается антагонистам минералокортикоидных рецепторов, как патогенетически обоснованным средствам при минералокортикоидном избытке, при необходимости в комбинации с блокаторами кальциевых каналов и другими препаратами, применяемыми для лечения артериальной гипертензии.

## 4. МЕДИЦИНСКАЯ РЕАБИЛИТАЦИЯ И САНАТОРНО-КУРОРТНОЕ ЛЕЧЕНИЕ, МЕДИЦИНСКИЕ ПОКАЗАНИЯ И ПРОТИВОПОКАЗАНИЯ К ПРИМЕНИНИЮ МЕТОДОВ МЕДИЦИНСКОЙ РЕАБИЛИТАЦИИ, В ТОМ ЧИСЛЕ ОСНОВАННЫХ НА ИСПОЛЬЗОВАНИИ ПРИРОДНЫХ ЛЕЧЕБНЫХ ФАКТОРОВ

Уровень убедительности рекомендаций C (уровень достоверности доказательств — 5)

Комментарии: рождение ребенка с неправильным строением наружных гениталий, т.е. с неопределенностью пола, часто является стрессом для семьи, что требует квалифицированной помощи психолога. Потребность в пожизненной заместительной терапии глюко- и минералокортикоидными препаратами и угроза развития острых состояний в виде кризов острой надпочечниковой недостаточности вызывают обеспокоенность как самих детей, так и их родителей. Психологическое сопровождение пациентов и членов их семей не только улучшает качество жизни пациентов, но и повышает приверженность к лечению. В подростковом возрасте у многих пациентов с ВДКН, перенесших пластическую хирургию на наружных гениталиях, возникают вопросы о половой принадлежности и о перспективах половой жизни и фертильности, что подчас требует занятий с психологом для принятия заболевания.

## 5. ПРОФИЛАКТИКА И ДИСПАНСЕРНОЕ НАБЛЮДЕНИЕ, МЕДИЦИНСКИЕ ПОКАЗАНИЯ И ПРОТИВОПОКАЗАНИЯ К ПРИМЕНЕНИЮ МЕТОДОВ ПРОФИЛАКТИКИ

## 5.1 Профилактика острых состояний

Профилактика при врожденной дисфункции коры надпочечников направлена на предотвращение кризов острой надпочечниковой недостаточности.

Уровень убедительности рекомендаций С (уровень достоверности доказательств — 4)

Уровень убедительности рекомендаций C (уровень достоверности доказательств — 4)

Комментарии: дети с классическими формами ВДКН имеют хроническую надпочечниковую недостаточность, следовательно, выброс достаточного количества глюкокортикоидов как реакция на стресс у них невозможен. Это диктует необходимость повышения дозы глюкокортикоидов в таких случаях, как оперативное лечение, лихорадка, обезвоживание или тяжелая травма. При интеркуррентных заболеваниях доза глюкокортикоидов увеличивается в 2–3 раза. При развитии адреналового криза требуется парентеральное введение глюкокортикоидов.

Уровень убедительности рекомендаций С (уровень достоверности доказательств — 5)

Комментарии: произвести парентеральное болюсное введение гидрокортизона** 50–100 мг/м² или из расчета 2–4 мг/кг. Расчет доз также возможен в зависимости от возраста пациента: в/м или в/в болюсно до 2 лет (<15 кг) — 25 мг, с 2 до 6 лет (15–25 кг) — 50 мг, с 6 лет и старше (>25 кг) — 100 мг гидрокортизона**. Далее продолжить введение гидрокортизона** в дозе 50–75–100 мг/м²/сут в зависимости от состояния либо в виде непрерывной инфузии, либо в виде четырех разделенных доз, вводимых каждые 6 часов внутривенно или внутримышечно.

Парентеральное восполнение жидкости:

- при шоке или средней/тяжелой дегидратации ввести в/в 0,9% раствор натрия хлорида** в дозе 10-20мл/кг

- начать последующую поддерживающую инфузионную терапию: 0,9% раствор натрия хлорида ** или 5% раствор декстрозы**

- при уровне глюкозы крови менее 3,5 ммоль/л — вводить в/в болюсно 10% раствор декстрозы** в дозе 2–5 мл/кг

Каждые 2 часа — исследование уровня калия, натрия, глюкозы в крови, а также измерение артериального давления на периферических артериях, измерение частоты сердцебиения.

При нормализации уровней калия, натрия — переход на в/м введение раствора гидрокортизона** с постепенным снижением дозы (1 мг/кг 1 раз в 6 часов) и последующим переходом на пероральные препараты. Флудрокортизон** назначают после перехода на пероральный прием при дозе гидрокортизона** <50 мг/м2/сут [89][90].

Уровень убедительности рекомендаций C (уровень достоверности доказательств — 5)

Комментарии авторов: при хирургических операциях с общей анестезией, тяжелых травмах, родах и заболеваниях требующих интенсивной терапии: непосредственно перед анестезией необходимо болюсное введение гидрокортизона** в дозе 2 мг/кг (максимально 100 мг) в/в или в/м (для недоношенных детей и детей младше 28 суток жизни в дозе 4 мг/кг) с последующим продолженным введением гидрокортизона** в/в капельно со скоростью введения из расчета:

- при массе тела менее 10 кг: 25 мг за 24 часа

- при массе тела 10-20 кг: 50 мг за 24 часа

- при массе тела более 20 кг: у допубертатных детей– 100 мг за 24 часа, у детей пубертататного возраста — 150 мг за 24 часа.

После завершения операции рекомендуется парентеральное введение гидрокортизона** в течение 12–24 часов в/в капельно в указанных выше дозах или с переходом на болюсное в/в или в/м введение гидрокортизона из расчета 1–2 мг/кг (максимально 50 мг) каждые 6 часов. На следующий день после оперативного лечения при стабилизации состояния, удовлетворительных показателях артериального давления и электролитов предпочтительно переходить на пероральный прием гидрокортизона** в стрессовых дозах (увеличить стандартную суточную дозу в 2 раза в течение 48 часов (из расчета до 30 мг/м²/сут) с последующим постепенным снижением дозы до обычной терапевтической дозы [90]. При переходе на пероральный прием гидрокортизона** и наличии у пациента минералокортикоидной недостаточности, рекомендовано назначить флудрокортизон** в стандартной дозе, получаемой пациентом до оперативного вмешательства.

## 5.2 Динамическое наблюдение (мониторинг терапии) при дефиците 21‑гидроксилазы

Целью терапии при ВДКН является подбор минимальной эффективной дозы глюкокортикоидов. Динамическое наблюдение пациентов с дефицитом 21‑гидроксилазы требует постоянного поддержания равновесия между избытком глюкокортикоидов, приводящим к развитию медикаментозного синдрома Кушинга с подавлением роста, избытком веса, повышением АД, и недостатком глюкокортикоидов, приводящим к развитию гиперандрогении с ускорением роста, опережением костного возраста и проявлением симптомов вирилизации.

Уровень убедительности рекомендаций C (уровень достоверности доказательств — 4)

Комментарии: дети первого года жизни должны наблюдаться с периодичностью 1 раз в 1–3 мес. Критерием адекватности заместительной терапии у грудных детей в первую очередь является кривая набора веса. Удовлетворительный набор веса, отсутствие срыгиваний свидетельствуют об эффективном лечении. Ежемесячно определяют уровни электролитов крови, по которым подбирается доза флудрокортизона**. Для активности ренина плазмы у детей младше 3 мес жизни не существует четких критериев, поэтому данный показатель использовать для оценки адекватной заместительной терапии минералокортикоидами сложно. Уровень 17ОНР определяют каждые 3 мес для подбора дозы гидрокортизона**. Следует отметить, что недостаточное подавление гиперандрогении на первом году жизни не приводит к существенной прогрессии костного возраста, тогда как передозировка глюкокортикоидов в этот период оказывает негативное влияние на конечный рост пациентов.

Уровень убедительности рекомендаций C (уровень достоверности доказательств — 5)

Комментарии: детей старше года детский эндокринолог должен осматривать 1 раз в 3–6 мес. Для этой возрастной категории среди критериев адекватности терапии на первый план выходят кривая роста и динамика прогрессии костного возраста. Снижение скорости роста свидетельствует о передозировке глюкокортикоидов. Увеличение скорости роста по сравнению с возрастными нормами и опережение паспортного возраста костным более чем на год за 1 год говорят о недостаточной дозе глюкокортикоидов. Целевым уровнем 17ОНР является верхняя граница нормы или несколько выше. У детей до начала пубертата и девочек-подростков информативным может быть определение уровня тестостерона, который отражает длительность существующей гиперандрогении. Небольшое повышение уровня 17ОНР при нормальных уровнях тестостерона не требует повышения дозы гидрокортизона**.

Уровень убедительности рекомендаций C (уровень достоверности доказательств — 5)

Комментарии: особого внимания требуют подростки с ВДКН, поскольку в этот возрастной период определяется будущая фертильность и реализуется ростовой потенциал. Передозировка глюкокортикоидов в это время приводит к конечной низкорослости пациентов. В связи с особенностями метаболизма кортизола в период пубертата очень сложно добиться адекватного подавления гиперандрогении, не вызывая при этом симптомов передозировки. Поэтому наблюдать за подростками с ВДКН необходимо 1 раз в 3–6 мес. В данной возрастной группе возможен переход на препараты глюкокортикоидов пролонгированного действия при условии наличия близких к закрытию зон роста и достижения конечного роста. Критериями адекватности терапии служат динамика роста и веса, а также скорость прогрессии полового развития. Как гиперандрогения, так и ятрогенный гиперкортицизм приводят к задержке полового развития.

В случае длительной декомпенсации заболевания необходимы дополнительные методы обследования. Чтобы исключить развитие вторичных аденом, проводится УЗИ надпочечников. У мальчиков дополнительно проводится ультразвуковое исследование органов мошонки с целью выявления гиперплазии эктопированной надпочечниковой ткани в яичках. Наличие вторичных аденом в надпочечниках или объемных образований в яичках требует ужесточения контроля гиперандрогении. В таких случаях или повышается доза гидрокортизона**, или пациент переводится с гидрокортизона** на препараты глюкокортикоидов пролонгированного действия.

Периоды длительной передозировки глюкокортикоидов могут вызывать осложнения, к которым относятся ожирение, гиперинсулинемия и инсулинорезистентность. В детском и подростковом возрасте данные осложнения не требуют дополнительного обследования, а при лечении, помимо коррекции глюкокортикоидной терапии, применяются диетотерапия и физические нагрузки.

Во все возрастные периоды, кроме грудного возраста, для коррекции минералокортикоидной терапии используется уровень активности ренина плазмы или уровень прямого ренина, который необходимо поддерживать на верхней границе нормы.

Длительная передозировка минералокортикоидов приводит к стойкой артериальной гипертензии и снижению уровня ренина. В таких случаях снижение дозы флудрокортизона** нужно проводить под контролем активности ренина плазмы или прямого ренина, т.к. нормализация артериального давления может происходить медленно и даже потребовать временного назначения гипотензивной терапии (используются блокаторы кальциевых каналов).

Повышение активности ренина плазмы даже при отсутствии электролитных изменений диктует необходимость увеличения дозы минералокортикоидов.

## 5.3. Динамическое наблюдение (мониторинг терапии) у детей с редкими формами ВДКН

Уровень убедительности рекомендаций C (уровень достоверности доказательств — 5)

## 6. ОРГАНИЗАЦИЯ ОКАЗАНИЯ МЕДИЦИНСКОЙ ПОМОЩИ

## Показания для госпитализации в медицинскую организацию:

## Показания к выписке пациента из медицинской организации:

## 7. ДОПОЛНИТЕЛЬНАЯ ИНФОРМАЦИЯ (В ТОМ ЧИСЛЕ ФАКТОРЫ, ВЛИЯЮЩИЕ НА ИСХОД ЗАБОЛЕВАНИЯ ИЛИ СОСТОЯНИЯ)

Как для любого моногенного заболевания, для всех форм врожденной дисфункции коры надпочечников возможно проведение пренатальной и преимплантационной диагностики в семьях, уже имеющих детей с данной патологией.

Уровень убедительности рекомендаций C (уровень достоверности доказательств — 4)

Комментарии: для проведения пренатальной диагностики необходимо знать генотип обоих родителей. Диагностика проводится на 9–10‑й неделях гестации путем получения ДНК из ворсин хориона и определения наличия мутаций гена, ответственного за развитие данной формы ВДКН.

Для ВДКН возможно проведение предимплантационной диагностики. Предимплантационное генетическое тестирование может проводиться только при применении вспомогательных репродуктивных технологий, а именно эктракорпорального оплодотворения (ЭКО) с интраплазматической инъекцией сперматозоидов.

Уровень убедительности рекомендаций В (уровень достоверности доказательств — 2)

Комментарии: пренатальная терапия проводится с целью избежать или снизить степень вирилизации наружных половых органов у плода женского пола с дефицитом 21‑гидроксилазы или 11в‑гидроксилазы. Для терапии применяется дексаметазон**, который не инактивируется плацентарной 11в‑гидроксистероиддегидрогеназой и проникает к плоду. Поскольку терапия должна начинаться с момента установления беременности, когда уровень кортизола у плода очень низкий, то в определенные периоды уровень глюкокортикоидов будет значительно превышать физиологический. Пренатальная терапия не позволяет избежать в дальнейшем пожизненной терапии и не является профилактикой сольтеряющих состояний в послеродовом периоде. Терапия показана только плодам женского пола с дефицитом 21‑гидроксилазы. Дефицит 21‑гидроксилазы является аутосомно-рецессивным заболеванием, и в семье, имеющей больного ребенка, вероятность беременности больной девочкой составляет 12,5%. Поскольку пренатальная диагностика возможна только на 10‑й неделе беременности, то в 87,5% случаев плод будет напрасно получать высокие дозы глюкокортикоидов с 4–6‑й до 10‑й недели гестации. Известны отрицательные последствия воздействия дексаметазона** на мать во время беременности: патологический набор веса, нарушения углеводного обмена и риск развития артериальной гипертензии. Не до конца изучены возможные эффекты воздействия высоких доз дексаметазона** на плод на ранних сроках гестации. Вследствие всего вышеизложенного пренатальная терапия не может быть рекомендована для внедрения в клиническую практику. Требуются дальнейшие исследования.

## ДОПОЛНИТЕЛЬНАЯ ИНФОРМАЦИЯ

Источники финансирования. Работа выполнена в рамках темы госзадания «Персонифицированная комплексная терапия задержки роста и полового развития у детей с врожденными орфанными эндокринопатиями», номер 126022417900-6

Конфликт интересов. Авторы декларируют отсутствие явных и потенциальных конфликтов интересов, связанных с публикацией на стоящей статьи.

Участие авторов. Все авторы одобрили финальную версию статьи перед публикацией, выразили согласие нести ответственность за все аспекты работы, подразумевающую надлежащее изучение и решение вопросов, связанных с точностью или добросовестностью любой части работы.

## Приложение А3.

Справочные материалы, включая соответствие показаний к применению и противопоказаний, способов применения и доз лекарственных препаратов инструкции по применению лекарственного препарата.

**Figure:**
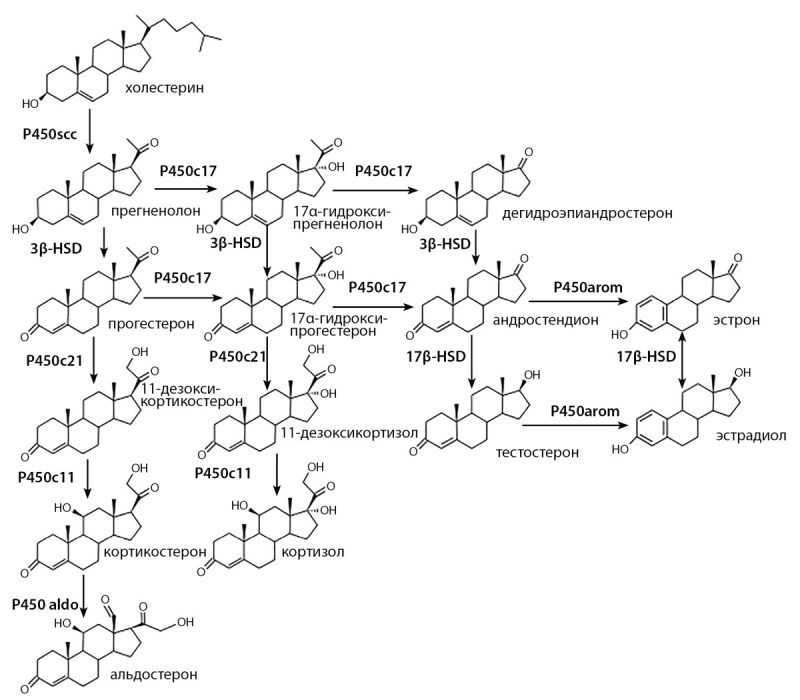
Рисунок 1. Схема стероидогенеза с указанием ферментов, участвующих в синтезе кортизола, дефекты которых приводят к врожденной дисфункции коры надпочечников.

## Приложение Б.

**Figure:**
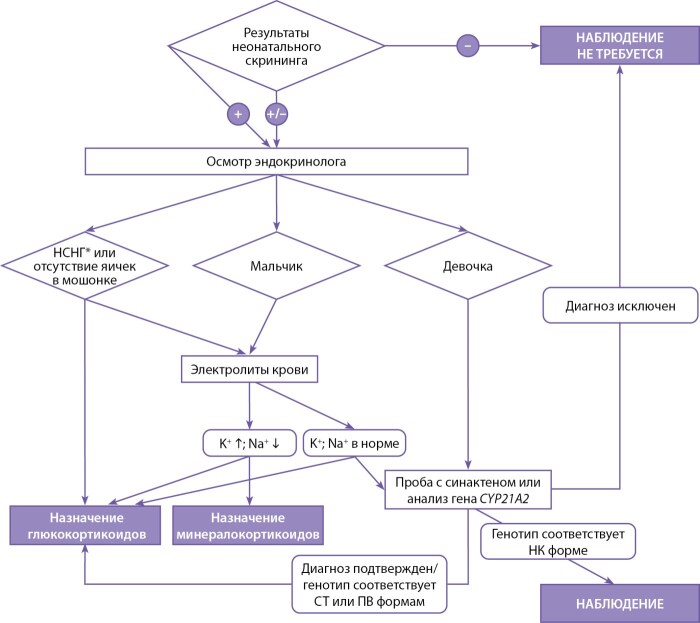
Рисунок 2. Алгоритм действий врача для диагностики и лечения врожденной дисфункции коры надпочечников у детей. Примечание: * НСНГ — неправильное строение наружных гениталий; СТ — сольтеряющая форма; ПВ — неклассическая форма.

## Приложение Г1.

**Figure:**
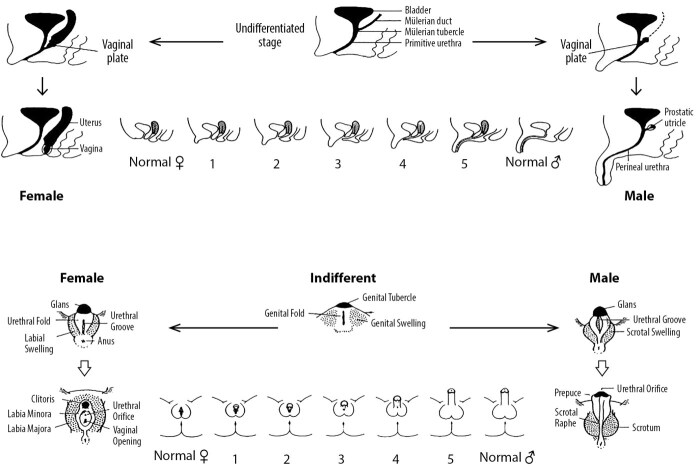
Рисунок 3. Шкала Прадера для оценки степени вирилизации наружных половых органов при врожденной дисфункции коры надпочечников. Источник: Prader A Der genitalbefund beim pseudohermaphroditismus femininus der kengenitalen adrenogenitalen syndroms. // Helv Paediatr Acta, 1954, N9, p.231-248 2 степень вирилизации — гипертрофия клитора и частичное сращение больших половых губ. 3 степень вирилизации — клитор гипертрофирован и сформирована его головка, сращение половых губ формирует урогенитальный синус — единое мочеполовое отверстие у основания клитора. 4–5 степень вирилизации — гипертрофированный клитор напоминает нормальный половой член, однако имеется его искривление (фиксация к промежности), урогенитальный синус открывается на стволе или головке полового члена (пинеальная уретра).
